# Novel pre‐spatial data fusion deep learning approach for multimodal volumetric outcome prediction models in radiotherapy

**DOI:** 10.1002/mp.17672

**Published:** 2025-02-10

**Authors:** John C. Asbach, Anurag K. Singh, Austin J. Iovoli, Mark Farrugia, Anh H. Le

**Affiliations:** ^1^ Jacobs School of Medicine and Biomedical Sciences State University of New York at Buffalo Buffalo New York USA; ^2^ Department of Radiation Medicine Roswell Park Comprehensive Cancer Center Buffalo New York USA; ^3^ Department of Radiation Oncology Cedar‐Sinai Medical Center Los Angeles California USA

**Keywords:** joint early pre‐spatial fusion, multimodal deep learning, neural network, outcome prediction, radiation therapy

## Abstract

**Background:**

Given the recent increased emphasis on multimodal neural networks to solve complex modeling tasks, the problem of outcome prediction for a course of treatment can be framed as fundamentally multimodal in nature. A patient's response to treatment will vary based on their specific anatomy and the proposed treatment plan—these factors are spatial and closely related. However, additional factors may also have importance, such as non‐spatial descriptive clinical characteristics, which can be structured as tabular data. It is critical to provide models with as comprehensive of a patient representation as possible, but inputs with differing data structures are incompatible in raw form; traditional models that consider these inputs require feature engineering prior to modeling. In neural networks, feature engineering can be organically integrated into the model itself, under one governing optimization, rather than performed prescriptively beforehand. However, the native incompatibility of different data structures must be addressed. Methods to reconcile structural incompatibilities in multimodal model inputs are called data fusion. We present a novel joint early pre‐spatial (JEPS) fusion technique and demonstrate that differences in fusion approach can produce significant model performance differences even when the data is identical.

**Purpose:**

To present a novel pre‐spatial fusion technique for volumetric neural networks and demonstrate its impact on model performance for pretreatment prediction of overall survival (OS).

**Methods:**

From a retrospective cohort of 531 head and neck patients treated at our clinic, we prepared an OS dataset of 222 data‐complete cases at a 2‐year post‐treatment time threshold. Each patient's data included CT imaging, dose array, approved structure set, and a tabular summary of the patient's demographics and survey data. To establish single‐modality baselines, we fit both a Cox Proportional Hazards model (CPH) and a dense neural network on only the tabular data, then we trained a 3D convolutional neural network (CNN) on only the volume data. Then, we trained five competing architectures for fusion of both modalities: two early fusion models, a late fusion model, a traditional joint fusion model, and the novel JEPS, where clinical data is merged into training upstream of most convolution operations. We used standardized 10‐fold cross validation to directly compare the performance of all models on identical train/test splits of patients, using area under the receiver‐operator curve (AUC) as the primary performance metric. We used a two‐tailed Student *t*‐test to assess the statistical significance (*p*‐value threshold 0.05) of any observed performance differences.

**Results:**

The JEPS design scored the highest, achieving a mean AUC of 0.779 ± 0.080. The late fusion model and clinical‐only CPH model scored second and third highest with 0.746 ± 0.066 and 0.720 ± 0.091 mean AUC, respectively. The performance differences between these three models were not statistically significant. All other comparison models scored significantly worse than the top performing JEPS model.

**Conclusion:**

For our OS evaluation, our JEPS fusion architecture achieves better integration of inputs and significantly improves predictive performance over most common multimodal approaches. The JEPS fusion technique is easily applied to any volumetric CNN.

## 1 INTRODUCTION

In recent years, interest in deep learning has significantly increased in many fields as neural networks prove powerful and adaptable at solving complex modeling tasks. Neural networks now dominate the field of computer vision, where certain model architectures, such as convolutional neural networks (CNN), can leverage iterative spatial processing of full image or volume data to embed a representation of key features at both the macro and micro scale. This makes deep learning particularly useful in various radiotherapy contexts, where spatial information is critically important. Neural networks that process spatial data have strict structure, requiring inputs to be uniformly shaped. Since a comprehensive representation of a radiotherapy patient spans multiple data modalities, alleviating this constraint and equipping models with more complete patient information is a critical pursuit. Techniques to accomplish this are collectively referred to as *multimodal fusion*. Generally, any modeling system which intakes data from multiple modalities and produces a unified output is multimodal modeling, but the decision of how to structurally combine the modalities’ information within the system defines the fusion itself. Understanding the importance of this decision motivates our work: we suggest that different fusion designs can meaningfully impact the performance of a multimodal model, even with identical inputs.

Early applications of deep learning computer vision in oncology primarily focused on diagnostic image evaluation and anatomical structure segmentation.^1^, ^2^, ^3^ This depends only on imaging inputs, avoiding the challenge of multimodal modeling. Neural networks were also trained on predictive tasks relevant to radiotherapy, such as predicting the expected dose‐volume histogram for treatment based on patient‐specific anatomy.^4^, ^5^ Arguably the most important prediction in radiotherapy is that of the patient's response to treatment, with overall survival (OS) of particular concern, for obvious reason. This problem space stands apart from those previously mentioned, since not only is OS prediction not primarily dependent on patient imaging, but many examples in literature find success without incorporating imaging data at all.^6^, ^7^, ^8^, ^9^, ^10^ However, there has also been research published demonstrating survival prediction models that do leverage imaging data.^11^, ^12^, ^13^, ^14^, ^15^ This suggests that information signaling a patient's probable outcome is present across multiple modalities. This observation, combined with the crucial importance of the task itself, lets OS prediction serve well as the task focus for multimodal data fusion exploration.

Although multimodal fusion is not a new concept, deliberate exploration of mechanics and design is fairly nascent. For classical machine learning algorithms, it is not usually feasible to customize the actual execution or architecture of the model, meaning that merging data modalities must either occur before the model, at the input feature level (early fusion), or after single‐modality models, at the prediction level (late fusion). These approaches are straightforward and well explored. However, neural networks allow precise control over the pathways of information flow, opening the door for multimodal fusion to occur *within* the model itself. This technique is less well explored; in fact, existing systematic reviews on multimodal fusion lack consistent language to describe it.^16^, ^17^, ^18^, ^19^, ^20^ In all cases, neural networks are performing feature extraction and producing an output prediction, both internal to the model architecture. If the only concern of a multimodal fusion is that each modality influences the output prediction, then the internal point‐of‐fusion is irrelevant. However, if cross‐modality information sharing might improve the feature extraction aspect of the model, then the point‐of‐fusion is a crucial consideration.

In this paper, we present a novel fusion technique, joint early pre‐spatial fusion (JEPS), and evaluate its performance against single modality baseline models as well as established multimodal techniques for 2‐year OS prediction on an internally sourced retrospective cohort of head and neck radiotherapy patients treated at our clinic. Our contributions are the following:

1. We introduce a novel approach to multimodal fusion that introduces nonvolume modality information upstream of most volumetric spatial processing. This allows supplemental, nonvolume data inputs to directly influence spatial feature extraction and regional emphasis.
2. We incorporate full spatial dose data derived from the planned treatment as an additional data channel in the volume input, laying the theoretical foundation for plan refinement feedback based on projected patient risk.
3. We perform extensive comparative experiments on single modality and multimodal models of various designs using a fixed data set, to include model interpretability analysis, in order to both demonstrate and understand the effectiveness of our approach.

### 1.1 Related work

Although synthesizing disparate imaging modalities is an active area of research, we distinguish our use of the term *multimodal* as referring to modalities that are *structurally incompatible*. Specifically, we explore combining spatial data, where the position of values carries implicit information in addition to the explicit information in the values themselves, with non‐spatial data, which has no implicit component. Systematic reviews of the subject agree on the terms *early fusion* and *late fusion*.^16^, ^17^, ^18^, ^19^, ^20^ *Early fusion* involves merging the modalities prior to performing task modeling, but for structurally incompatible data, this requires feature extraction to reduce the data to similar form. One of the most common and successful approaches in literature is the use of radiomics to convert annotated spatial data into a one‐dimensional array of non‐spatial features.^7^, ^8^, ^21^, ^22^, ^23^, ^24^, ^25^ Some research has explored using an independent neural network to convert the spatial data into a one‐dimensional representation compatible with non‐spatial data.^26^ In any case, early fusion involves fixed feature extraction that does not have access to non‐spatial information; in many ways, this is essentially preprocessing upstream of the actual modeling. *Late fusion* requires independent task models for each modality, and the predictions of these models are synthesized in some way to produce a single collective prediction representative of the multimodal system. This is mechanically similar to the model ensemble, a technique whose history traces as far back as the 1980s.^27^ In 2020, Huang et al. published work on multimodal pulmonary embolism detection, including a good representation of various late fusion approaches, with their “Late Elastic Averaging” fusion, scoring highest of all approaches compared.^28^ Late fusion is even more problematic in terms of information sharing, since each individual modality's prediction is generated independently with no cross‐modality information exchange.

This leaves fusion that is between early and late: fusion occurring within the model itself. Classical machine learning is largely incompatible with this approach, and neural networks’ rise to prominence has enabled its exploration for the first time. Language for this technique is not standardized, but it has been called *intermediate*, *hybrid*, or *joint* fusion.^17^, ^19^, ^28^ The key feature of this type of fusion is the single governing loss function: since the different modalities are both provided to the one model and fed forward to a unified output, the backpropagation of loss gradients allows simultaneous adjustment of weights across all modalities during the model training process. Within joint fusion research, the question of the impact of point‐of‐fusion is underexplored. In spatial networks, such as CNN, tensor shape throughout the model is a critical architectural aspect. Terminology to differentiate model‐internal point‐of‐fusion choices is lacking, but throughout literature, fusion of spatial and non‐spatial data occurs late in the model architecture, after the spatial data path has been fully featurized into a one‐dimensional tensor.^28^, ^29^, ^30^, ^31^, ^32^, ^33^, ^34^ With this approach, a heavy burden is placed on each modality's backpropagation of loss gradients, as the only way cross‐modality information sharing can enhance feature extraction is indirectly. We sought to explore more direct information sharing during the spatial feature extraction portion of the neural network architecture. In our literature search, we found no published work exploring this type of pre‐spatial fusion, making our JEPS approach novel in this domain.

## 2 METHODS

### 2.1 Model designs

#### 2.1.1 Single modality models

Although comparing JEPS to established multimodal strategies was our main focus, we also trained several baseline models on single‐modality data. Survival analysis is commonly performed with regression‐based models, such as the Cox Proportional Hazards (CPH) model.^35^, ^36^ We chose a CPH model to represent our non‐deep learning baseline on clinical inputs only. Then, since our focus is on deep learning, we designed a simple multi‐layer perceptron (MLP) model to also train only on clinical data. This model was made up of three fully connected hidden layers with 64, 32, and 16 neurons, in that order. Some existing literature exploring spatial neural networks in this space forego use of supplemental data.^11^, ^15^, ^37^, ^38^ To baseline the performance of a model with only volume input, we built a 3D CNN using a ResNet‐18 design structure. This model had 2 094 341 total parameters.

#### 2.1.2 Fusion models

As discussed, there are three primary categories of fusion models: early, late, and joint fusion. We represent each category in our experiment. For early fusion, which combines the two modality data arrays prior to passing them to the model, we extracted radiomics features for tumors in each patient volume, filtered out low variance fields from the result, then concatenated these values with the preprocessed array of clinical input data, creating a multimodal input array. We then trained the same model types as were used on the only clinical data input: CPH and MLP. The configurations for these models were the same; the only change was the multimodal input instead of the single modality input. To represent late fusion, where each modality is passed through an independent model and the outputs are synthesized, we arranged our single modality models to produce independent outputs and averaged the two results into a fused multimodal prediction.

**FIGURE 1:**
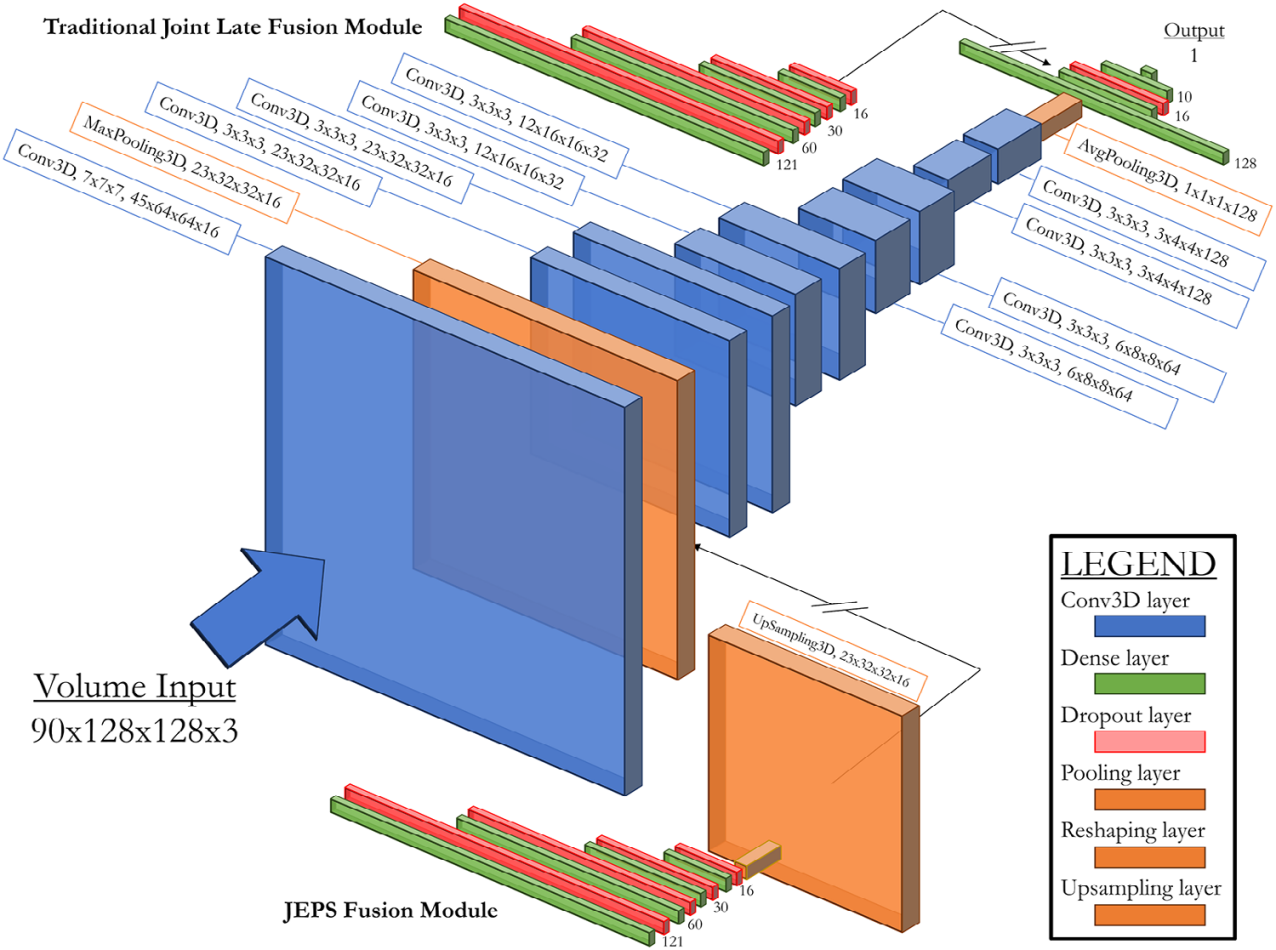
Diagram of CNN architectures. Baseline ResNet‐18 is shown in the center, while the joint late fusion and joint pre‐spatial fusion modules are visible above and below, respectively. CNN, convolutional neural network.

The two joint fusion models—a traditional joint late fusion approach and our novel JEPS architecture—were both adapted from the baseline single modality ResNet architecture used for volume‐only input data. Figure 1 shows a diagram of this baseline ResNet model architecture, as well as the design of the supplementary fusion modules. For the joint late fusion model, we followed common practice in literature and kept the data processing separate for our two inputs until after all spatial embedding of the volume was complete, joining the two data flows once the volume embedding was flattened into a 1D tensor. For our JEPS model, we placed the fusion point after the very first convolutional and pooling layer, upstream of the majority of the spatial embedding. To enable this, the early fusion module has an additional upsampling step. This upsampling step is of critical importance. In practice, this upsampling operation first receives a length‐16 tensor of the clinical data embedding and adds three dimensions to it, resulting in a 4D tensor with a shape of 1 × 1 × 1 × 16, where the embedding resides in the channels axis. Then, this 4D tensor is repeated using the Keras package's *UpSampling3D* layer so that its shape matches the shape of the volume tensor at the point of fusion—23 × 32 × 32 × 16. This allows direct concatenation of the clinical data tensor onto the volume tensor. Rather than dilute the information signal or inadvertently assign some spatial significance via a partial projection, the clinical data embedding is applied equally across the volume tensor. We rely on subsequent convolutional layers, operating on the fused multimodal representation, to organically determine which spatial regions should have strong clinical information retention and which should not. Apart from the upsampling, both early and late fusion process the clinical data identically, made up of fully connected layers, with incremental reductions in size, interleaved with dropout layers to avoid individual feature overreliance. At the end of this process, the clinical data input is embedding into a tensor 16 values long. In late fusion, this tensor can simply be concatenated with the embedding of the volume data, since the volume data embedding has also been embedded into a flat, length‐16 tensor by that point in the architecture. In early fusion, however, the receiving tensor of volume data still has a 4D shape, and the upsampling projects the clinical data embedding into an identically shaped volume for concatenation in the channel's axis. This highlights the nature of the innovation: the data is identical, and the core processing elements are identical, so the only possible explanation for any performance differences is the differing fusion strategies. The addition of either fusion module only slightly increases the total model size: the joint late fusion design has 2 104 243 parameters, and the JEPS fusion has slightly more with 2 111 427, due to the additional upsampling.

### 2.2 Patient data

An initial patient cohort of 531 patients was retrospectively sourced from internal records of patients with head and neck cancer who completed treatment at our clinic between 2014 and 2021. While imaging and dose data are typically retained, standardized collection of both clinical attribute data and patient follow‐up data required coordinated effort across providers over many years. For each patient, we first exported from the Varian Eclipse treatment planning system (version 16.0.0) the following DICOM files: all planning computed tomography (CT) images, structure set file, and dose file. These files formed the basis of the volume data input to our models. Each CT study was acquired by helical scan with a large‐bore GE Discovery CT simulator (GE Healthcare, Chicago, IL, USA) using a head and neck protocol of 120 kVp, auto mA, large field of view, 2.5‐mm axial slice thickness, and 512 × 512  pixel resolution. Separately, follow‐up data was retrieved. This was stored in and exported from a REDCap database managed internally at our clinic.^39^, ^40^ Patients missing data were excluded, leaving 407 patients, 80 of which were deceased. To simplify the modeling, we chose a binary classification of 2‐year survival, sourced from follow‐up data, as our endpoint. To avoid right‐censored data, living patients without follow‐up beyond 2 years post‐treatment were excluded. This provided a concrete, objective ground truth for each case. The final cohort was 222 patients, 63 of which died within 2 years of treatment completion.

For the supplemental tabular data input, we gathered clinical data that had been collected at patient intake which was also stored in a REDCap database. A wide range of fields exist in the clinical database, representing things like patient demographics, medical history, lifestyle information, and more. We removed fields with low variance and all free text fields, then selected a subset of 28 fields that represent a mix of demographic data, cancer and treatment details, and other existing medical conditions. Table 1 shows the distributions of the patient cohort. Importantly, no data in any input modality was sourced from mid‐treatment or post‐treatment collections, making any resultant models capable of performing pre‐treatment evaluations.

**TABLE 1:** Patient cohort distribution for clinical data fields included in model input.

Patient summary					
**Demographics**			**Conditions**		
Age at diagnosis (years)	Mean	61.97	HPV status	Positive	121
	IQR	13.57		Negative	47
Gender	Male	178		Unknown	54
	Female	44	Alcohol	Current	107
Marital status	Married	123	consumption	Former	36
	Single	62		Never	22
	Divorced	18		Not reported	57
	Other/Unknown	19	Current smoking	Current	37
**Physical**			status	Former	122
Height (cm)	Mean	172.59		Never	63
	IQR	11.8	Nutrition support	Yes	79
Weight (kg)	Mean	84.83		No	143
	IQR	27.70	**Other conditions**		
BMI	Mean	28.37	Respiratory	Yes	50
	IQR	7.42		Not reported	172
**Cancer details**			Nervous	Yes	52
T stage clinical	T0	19		Not reported	170
	T1	40	Endocrine	Yes	43
	T2	58		Not reported	179
	T3	70	Cardiovascular	Yes	139
	T4	35		Not reported	83
N stage	N0	37	Digestive	Yes	56
	N1	43		Not reported	166
	N2	25	**Medications**		
	N2a	10	Anti‐coagulants	Yes	26
	N2b	60		No	196
	N2c	29	Anti‐inflammatory	Yes	95
	N3	18		No	127
Overall clinical stage	1	16	Anti‐convulsants	Yes	24
	2	11		No	198
	3	47	Anti‐anxiety/depr.	Yes	51
	4	148		No	171
Disease site	Pharynx	161	Narcotic Analgesics	Yes	50
	Larynx	39		No	172
	Other	22	Vitamins/Minerals	Yes	102
Treatment type	CCRT	179		No	120
	ICT + CCRT	22			
	RT only	14			
	Surgery + CCRT	7			
Unilateral or bilateral	Bilateral	142			
	Unilateral	62			
	Not recorded	18			

Abbreviations: BMI, body mass index; CCRT, concurrent chemoradiation therapy; HPV, human papillomavirus; ICT, immune checkpoint therapy; N stage, lymph node stage; RT, radiotherapy; T stage, tumor stage.

This retrospective collection and de‐identification of patient data was performed in compliance with our institutional ethical standards and with the 1964 Helsinki declaration. This retrospective study was conducted under institutional review board approval (No. EDR‐103707).

### 2.3 Data processing

The CT images were stacked based on the axial position metadata, then resampled via spline interpolation into voxels sized 2 × 2 × 2.5 mm. The dose array was resampled in the same way, then overlaid, using DICOM metadata to ensure voxel alignment. This created a fourth dimension, referred to as the “channels axis.” For the mask channel, each point within the structure set contour for the planning target volume (PTV) was mapped to the nearest voxel position in the array. We then performed slice‐wise contour filling using the OpenCV software package's “fillPoly” function, resulting in a binary mask of each slice's tumor volume.^41^ Since tumor locations varied across the dataset, we used the parotid gland contours as a positional reference to standardize the anatomy in the patient volume, cropping each volume to a size of 128 × 128 × 90 × 3 voxels with the center axial plane passing through the axial center of the parotid glands. This eliminated many extraneous voxels and improved standardization of patient anatomy presentation.

The CT voxels were first converted into Hounsfield units, then a window/level filter of 400/50 was applied to the CT channel, a typical range for tissue contrast. In order to ensure all inputs fell within the same range, the CT voxel values were rescaled to between 0.0 and 1.0 from their post‐W/L filter range of –150 to 250 using min‐max standardization. The dose channel, originally in Gray, was similarly rescaled, using 0.0 to 70.0 as the range for min‐max standardization, since 70  Gy was the greatest prescribed dose in the cohort. Lastly, all patients whose ipsilateral side was their left side were mirrored about the sagittal plane to reduce variance in tumor presentations for modeling. For the early fusion models, which require a unified 1D input, we used the Python package *pyradiomics* to reduce the volume to radiomics features.^42^ We performed this extraction on the volumes after aligning and resampling them, but before performing any voxel value transformations. We used the standard configuration of *pyradiomics* to generate a suite of features, then incorporated a filter at train time that standardized each feature via min‐max standardization to between 0.0 and 1.0, then dropped any feature with calculated train set variance below 0.05. Because we used cross‐fold validation, performing this filtering at train time was critical to ensure the preprocessing was only governed by the training data, since performing it on the total dataset would introduce implicit information leakage across train and test sets.

**FIGURE 2:**
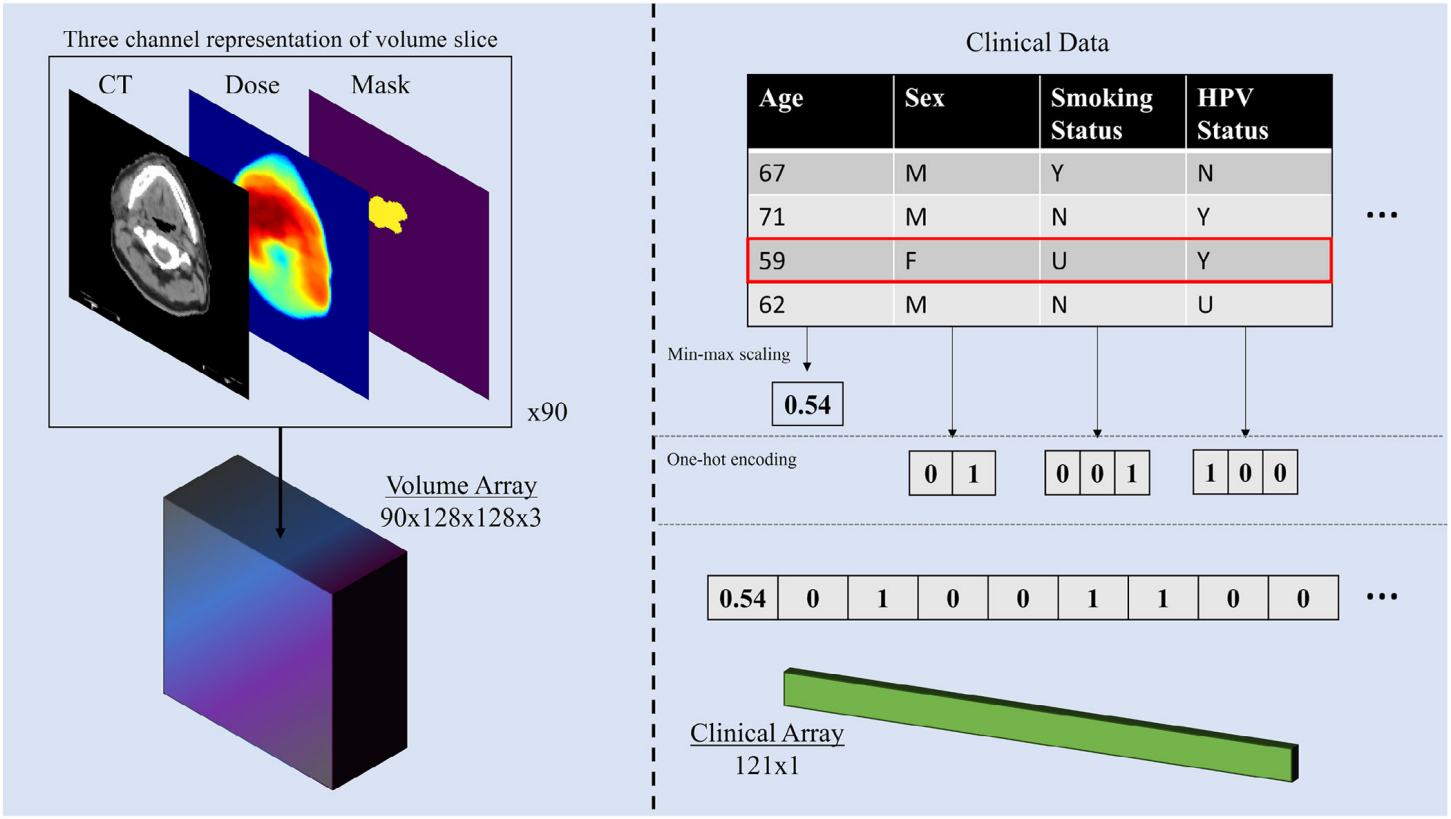
Visualization of preprocessing workflow. Spatial data was aligned on a slice‐wise basis and stacked into a volume array with three information channels. Clinical data was processed according to type (min‐max scaling for numeric variables, one‐hot encoding for categorical variables) and stacked into a one‐dimensional array.

For clinical data, numeric fields underwent min‐max standardization scaling to between 0.0 and 1.0, while categorical features were one‐hot encoded. Importantly, since cross‐validation was used during model training and evaluation, the clinical data preprocessing was performed on a per‐fold basis during cross‐validation, not on the dataset as a whole. This allowed the numeric field scaling range to be derived from only the training data for each iteration, preventing indirect information leakage. For one‐hot encoding, this means that any field values present in test data that were not represented in training data would not be captured—the array for that field would be only zero values. Figure 2 visualizes the data preparation process for both input modalities.

On‐line data augmentation of patient volumes was implemented to minimize overfitting. During iterative model training, each patient volume in each batch had a 2/3 chance of undergoing data augmentation and 1/3 chance of unmodified throughput. Three augmentation operations were possible, randomly determined—zoom at 0.8–1.2 factor, rotation up to ± 15 degrees in the axial plane and shift up to 20% in any direction except axially. No augmentation was performed on clinical data, so models which did not use volume data had no data augmentation.

### 2.4 Training strategy

Because of the high dimensionality—and therefore increased modeling complexity—of the inputs, we chose a k‐fold cross validation data strategy to maximally leverage our data set. This provides a more effective evaluation method than separating a single holdout test dataset while still ensuring train/test data segregation for each training instance.^43^ We split the dataset into 10‐folds, or splits, of comparable size with representative distribution of positive and negative cases. For each of the models evaluated, 10 distinct versions of the model were created, one for each split. Each model instance would be tested on its assigned data split, and it would train on the remaining nine splits. From within the training data, 10%, randomly selected, was set aside each training run to serve as validation data. Note that in any case where selections were made randomly, such as validation data or data augmentation regimes, the script included prescriptive seeding of the random number generator. This made data preparation for a given split reproducible across all models, ensuring fair comparisons in evaluation.

Each neural network was trained using similar settings. Each model used the Adam optimizer and monitored binary cross‐entropy as the loss function.^44^ Due to the class imbalance, positive cases were overweighted at a 2:1 ratio against negative cases. Overfitting was mitigated by data augmentation for the volume inputs, as mentioned previously, and dropout layers for the tabular inputs. The batch size was 25 patients, and the initial learning rate was 0.001 with scheduled reductions. Early stopping ended training if validation loss did not decrease for 60 epochs. Notably, we did not perform aggressive hyperparameter tuning, since the competing model architectures might benefit unequally from it, and we preferred to use consistent hyperparameter settings for each model to not confound the performance comparisons.

### 2.5 Evaluation

The primary quantitative metric for evaluating the performance of our classifier was area under the receiver‐operating characteristic curve (ROC), or area‐under‐curve (AUC). AUC provides a more granular view of model performance by not requiring output binarization, while also being a suitable metric for class‐imbalanced datasets such as ours. Because our cross‐validation strategy produced 10 instances of each model, we scored each instance of each model on its test set, and these 10 scores formed a population of scores for that model type. Since each model for any given fold was trained and tested on the same data across model designs, we performed statistical analysis of the per‐fold results via a paired, two‐tailed Student *t*‐test to assess the significance of performance differences. For this analysis, our null hypothesis for each comparison was that there was no systemic difference in model performance due to architecture changes, and any observed difference in population mean AUC was due to random chance. We used a *p*‐value threshold (alpha) of 0.05, so scores below this threshold allowed us to reject the null hypothesis and consider the performance differences for that compared pair to be statistically significant. Although 10‐fold cross validation produces relatively small populations for statistical testing, the paired Student *t*‐test still provides robust evaluation against false positives even with small sample sizes.^45^, ^46^ Depending on effect size, the statistical power and false negative rate of this test will vary.^47^ Because of this, when evaluating the results of the statistical tests, we can have confidence in reported significance, but we must be cautious not to assume too much meaning from a failure to reject the null hypothesis.

For model interpretability, we used Shapley value analysis on our models.^48^ This analysis determines an expected value for each position in each input, based on the model's training data, then evaluates the change in model prediction when an expected value is replaced by the real value from a test patient's data. In this way, the analysis iteratively assigns an “influence” score to each individual value in each input of each test patient. By aggregating these values, we can obtain some understanding for the mechanisms of the model's consumption of the inputs.

## 3 RESULTS

The individual test fold AUC scores for each model design can be seen in Table 2. The best performing model by mean AUC was our novel JEPS design, which scored 0.779.

**TABLE 2:** Fold‐wise AUC results for each model design.

Cross‐validation ROC AUC performance summary								
	Clinical only		Volume only	Fusion models				
Fold	CPH	MLP	ResNet	Late fusion	Early fusion		Joint fusion	
				Avg	CPH	MLP	Joint late	JEPS
*1*	0.723	0.652	0.696	0.768	0.411	0.643	0.589	0.705
*2*	0.955	0.786	0.714	0.830	0.455	0.732	0.679	0.857
*3*	0.696	0.598	0.688	0.688	0.946	0.580	0.786	0.759
*4*	0.854	0.740	0.677	0.771	0.646	0.700	0.594	0.729
*5*	0.667	0.708	0.688	0.750	0.365	0.781	0.792	0.792
*6*	0.490	0.531	0.656	0.615	0.677	0.562	0.594	0.635
*7*	0.771	0.646	0.635	0.677	0.646	0.700	0.771	0.844
*8*	0.750	0.573	0.635	0.635	0.083	0.573	0.448	0.615
*9*	0.613	0.844	0.500	0.812	0.229	0.875	0.677	0.896
*10*	0.678	0.822	0.689	0.911	0.244	0.822	0.722	0.956
**Mean **± CI95	**0.720 **± 0.091	**0.690 **± 0.077	**0.658** ± 0.044	**0.746 **± 0.066	**0.470 **± 0.185	**0.697 **± 0.078	**0.665 **± 0.079	**0.779 **± 0.080

*Note*: Mean fold‐wise values (bold) with CI95 are presented. Confidence intervals are calculated using Student *t* distribution.

Abbreviations: AUC, area under the receiver‐operator curve; CI95, 95% confidence intervals; CPH, Cox proportional hazards; JEPS, joint early pre‐spatial; MLP, multi‐layer perceptron.

**FIGURE 3:**
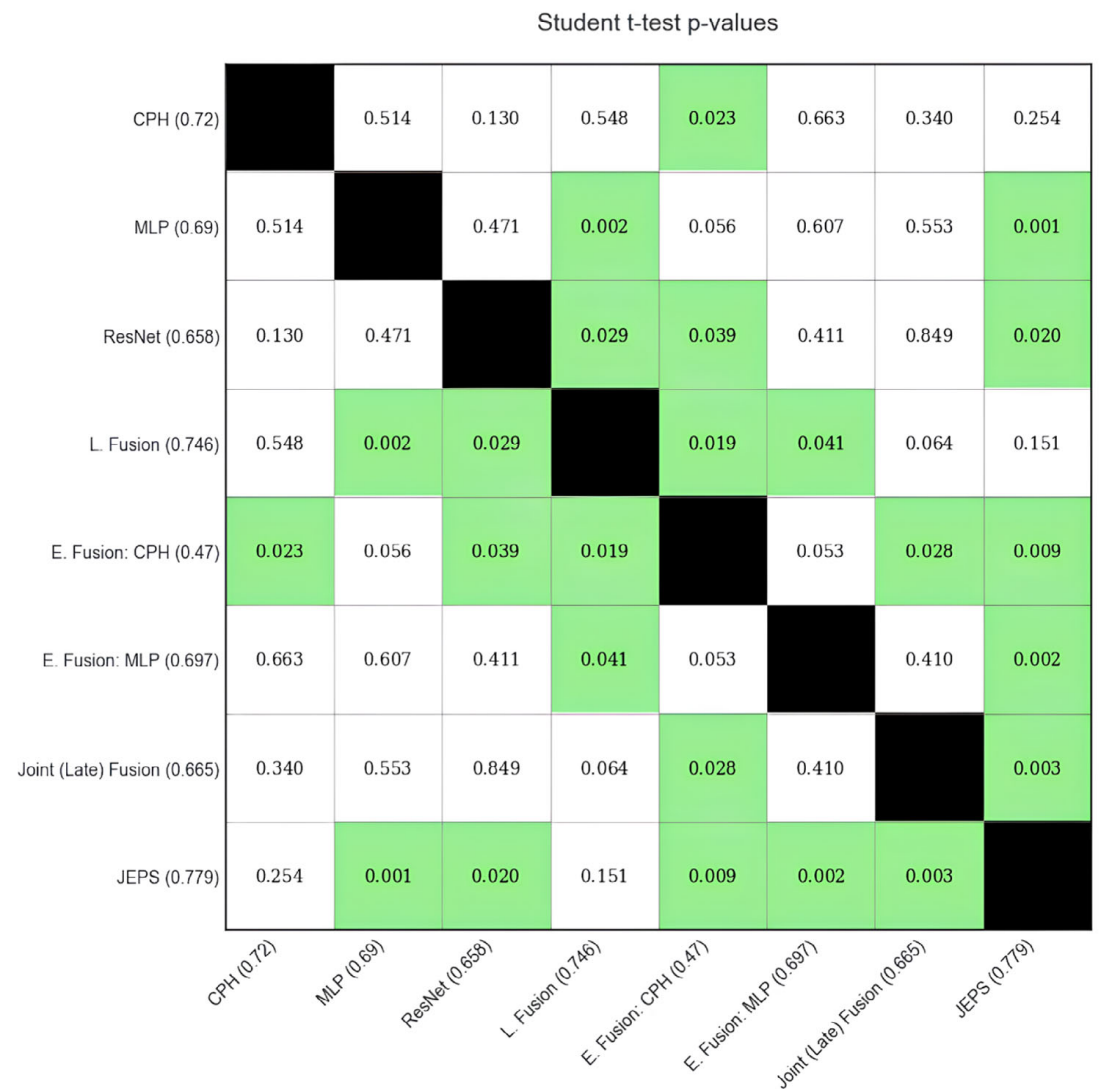
Pairwise comparison matrix of dependent Student *t*‐test significance results. Models are indicated by descriptive name followed by mean ROC AUC score in parentheses. Green highlights indicate a statistically significant difference.

Figure 3 shows the results of the Student *t*‐test statistical analysis. While our JEPS technique achieves the highest mean AUC, two other models score nearly as high: the single modality CPH model, and the late fusion model. None of these three models emerge as significant from one another; however, the JEPS model performs significantly better than all other tested models, while the CPH and late fusion model have, respectively, four and one other models that they do not significantly outperform.

**FIGURE 4:**
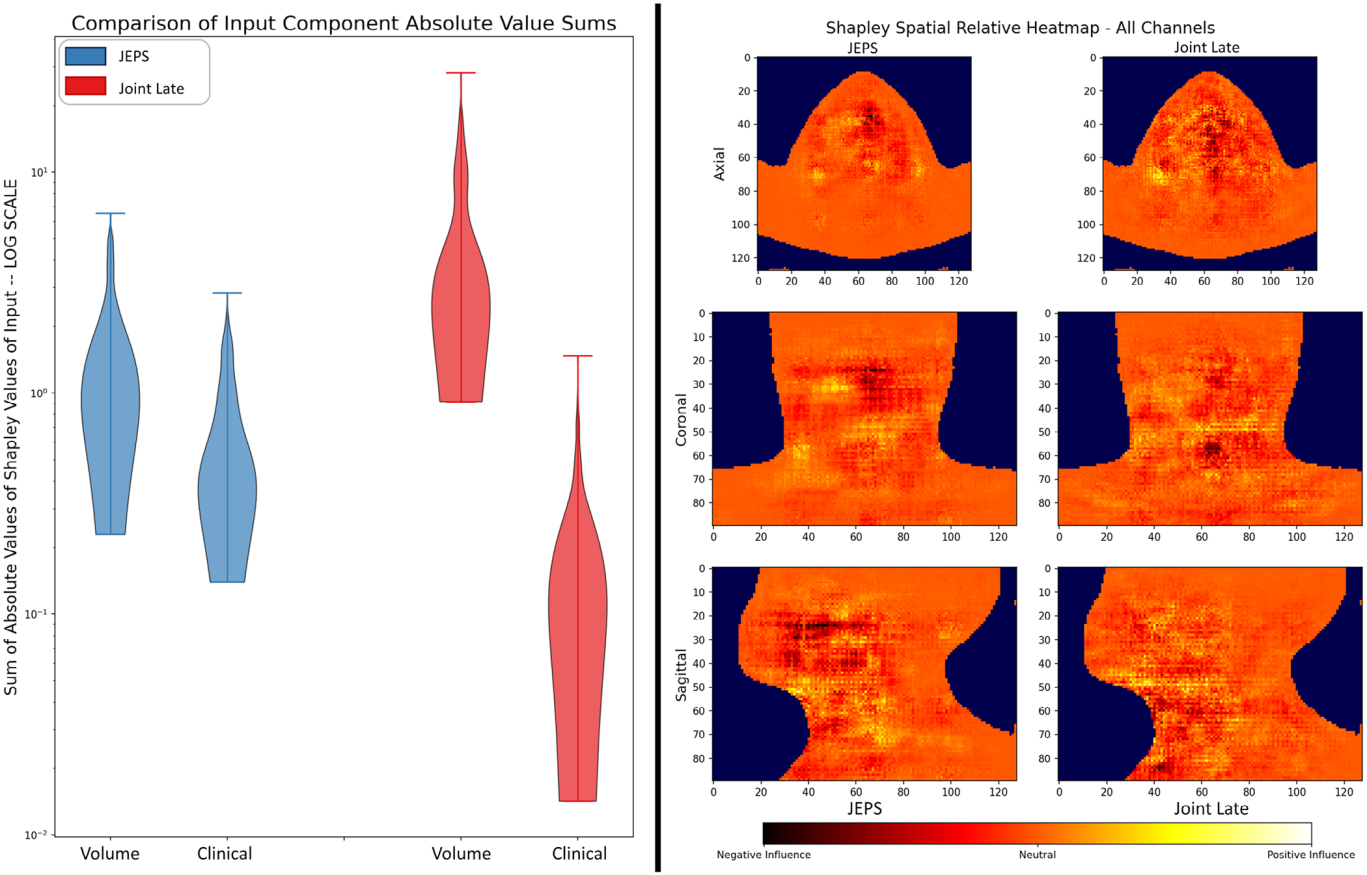
Per‐modality summed absolute value Shapley comparison violin plot for joint fusion models (left) and spatial heatmap of relative voxel‐level Shapley values in tri‐plane view (right). Note that for easier viewing the heatmaps are not scaled against one another; each heatmap is color scaled independently.

To better understand the significant performance difference between the joint fusion techniques tested, we turn to the Shapley analysis. Figure 4 shows the result of the Shapley value analysis both in absolute value aggregate, to assess the relative overall influence of each input modality for the joint fusion approaches, as well as in the form of a spatial relative heatmap representing the average Shapley value for each voxel position across the entire dataset (all folds). Figure 4 shows this combined volume from three views, each one flattened along a different axis. Although the heatmaps are independently scaled for improved viewability, they still reveal localized trends in voxel influence. Qualitative review by visual inspection shows the JEPS heatmap to be comparatively smoother, increasing the explainability of the model. In the JEPS heatmap, we see in the axial and coronal view a clear “triangle” of hot spots. These spots approximately encircle the location of highest frequency tumor occurrence in our dataset. This is an intuitive and understandable result. Voxels which frequently represent tumor would have less discriminatory value, whereas voxels on the lower frequency margins are of higher importance: tumor presence there may correlate in the training data to particularly advanced tumor progression, and the model updates its estimation of patient survival accordingly. Since these Shapley values account for all channels of the voxels, other hot or cold spots might be the result of anatomy, dose distribution, or tumor presence as annotated by the PTV mask channel. In the joint late fusion heatmap, we see less smooth importance gradients from region to region, making it more challenging to theorize the model's “logic” in semantic terms.

## 4 DISCUSSION

Our primary aim of this work was to evaluate the hypothesis that the point‐of‐fusion of a joint fusion multimodal neural network could significantly impact the performance of the model. In this, we were successful. Not only did we find our novel JEPS technique significantly outperformed the more traditional joint late fusion, but the JEPS model was the single highest performing model by mean AUC of all models evaluated. This is a promising result. We see from the performance of the single modality models that each modality contains some information signal meaningful to the task. If we assume the information signal across modalities is not redundant, where key signal in both modalities would just be different representations of the same indicators, then approaching the problem with multimodal modeling is well justified. This is supported by the results, where the two best performing models were multimodal. Within the joint fusion subset, the superior performance of our JEPS architecture over the joint late fusion architecture is important to emphasize due to the prevalence of joint late fusion in literature as the presumed design for joint fusion experiments. In presenting these results, we make the case for further stratification of joint fusion model architectures.

To help understand critical differences in the modeling approaches, we can view the model comparisons through an information theory lens. Each model considered has some informational shortcoming that joint fusion aims to address: (1) single modality models lack access to information from the absent modality, early fusion models lose spatial context and dose volume information, and (2) late fusion prevents cross‐modality information sharing during modeling. Joint fusion addresses these shortcomings by taking a full representation of each modality without information loss and allowing cross‐modality information exchange within the model, under the supervision of the model's single loss function and optimizer. However, within the architecture of a neural network, there are still information transformations. Since the joint late fusion does not merge modality tensors until after the volume input has been featurized, the clinical data is less able to influence the featurization of the volume. The model's output will have access to both modalities' information for prediction, but during optimization, the only avenue for one modality to influence the featurization of the other is extremely indirect: we must hope that one modality's data will assert influence on the prediction and loss function that will be subsequently felt via backpropagation by the other modality's featurization steps. The JEPS technique, on the other hand, overlays the clinical information onto the volume information prior to featurization, meaning that throughout the processing of the volume, the model has direct access to the clinical information to weigh the featurization against. This superior integration of modalities for both prediction *and* featurization is a possible explanation for the performance difference observed.

The Shapley value analysis provides additional insight into the difference between the joint fusion models by demystifying the “black box” of the deep learning architectures. Specifically, the violin plot in Figure 4 shows the aggregate influence of the distinct input modalities for each model architecture, and it is very obvious that the JEPS architecture does a much better job balancing the two inputs. Since the *y*‐axis in the violin plot is logarithmic scale, we observe that the joint late fusion architecture is more sensitive to the volume input by about an order of magnitude, while the JEPS is approximately equally sensitive, generally. This suggests that our JEPS design, by fusing the two inputs prior to most of the convolutional operations, is better able to develop key features that take into account information from both inputs. In problem spaces where one believes the non‐volume data to have important discriminatory value, such as for our problem of OS prediction, better ability to incorporate such inputs are valuable and important. The Shapley value observation could also explain the similarity in performance between the joint late fusion and the volume‐only ResNet model. According to the Shapley analysis, the joint late fusion model is dominated by the volume input. Given that treatment prognosis in clinical settings is usually not driven by a whole‐volume analysis, but clinical variables are nearly always considered, the model performance differences match our intuition based on the Shapley analysis results—specifically, we expect that, between two models, the one that better incorporates clinical data should perform better.

Our experiment has two limitations, and it is important to differentiate our work, which presents a novel modeling *technique*, from more mature works which may emphasize the models themselves. A critical limitation of our study is the makeup of the dataset—both its size, and its sourcing. A single‐center dataset specifically limits our ability to assess the model's generalizability. While we may demonstrate a performant model, we have no assurance that the key features the model leverages for its predictions are universally relevant to head and neck cancer patients instead of specific to our center's patient population. The only way to address this concern would be to source data from multiple centers, and this task would be a prerequisite to any meaningful effort to optimize or formally implement the JEPS technique. Additionally, the dataset is small. While this is a frequent problem in medical modeling, joint fusion neural networks are significantly more complex, exacerbating the issue. In this way, the limitation is not just of our experiment, but a core limitation of joint fusion and, by extension, our JEPS approach. We feel confident that the cross‐validation approach to model evaluation was the best possible use of the dataset available to us, and we are confident in our results. Nevertheless, the need for more data to properly leverage the more complex and powerful structure of JEPS should be viewed as a practical *disadvantage* of the approach.

Another limitation of our work is the lack of thorough optimization of the models presented. In order to simplify the direct comparison, which was critical to the experiment, we wanted the models to be configured as similarly as possible, so that no performance difference could be attributed to hyperparameters. Due to this, we sought configurations that provided stable, but not necessarily optimized, performance. In practice, the different model architectures might benefit from independent hyperparameter tuning, and exploring the impact of model hyperparameters on the models evaluated would be an important addition to this work. Beyond hyperparameter tuning, we also presented a simple neural network as our core volume processing model: a traditional ResNet. Many innovations in computer vision have been developed in recent years, such as attention‐based models. Expanded literature search outside of the medical domain shows that explorations of other such techniques for multimodal fusion has precedent.^49^ The attention mechanism offers some unique and potentially valuable insights. While our ResNet based model relied on coarse Shapley analysis to examine subregion importance and had no straightforward way to evaluate the relationships between clinical data and particular subregions, a mixture of self‐attention and cross‐attention between modalities could, if successful, serve as a more interpretable “map” of the relationships between key data. Ultimately, since attention mechanisms operate on sequences, not spatially, they require prior patch extraction and featurization within the model architecture. Nothing about the attention mechanism precludes the application of JEPS fusion techniques. Its most well‐known use is the Vision Transformer (ViT) design, where attention is paired with patch encoding of the volume data to perform spatial processing. A ViT model with JEPS fusion would simply require the fusion to occur prior to the patch encoding and attention layers. However, since our work focused on introducing the JEPS technique and contextualizing it within the current state of the field, we elected to only examine a more traditional CNN architecture due to its strong representation in existing literature. Having demonstrated the viability of the concept of pre‐spatial fusion, adapting it to more advanced architectures would be a very compelling future expansion of the research.

## 5 CONCLUSIONS

In this paper, we have described and implemented a novel JEPS fusion approach to incorporating tabular data inputs into volumetric computer vision neural networks. We have shown that our novel approach leads to significantly better model performance than traditional joint fusion methods on the task of OS outcome prediction. Since the nature of radiotherapy is fundamentally multimodal, methods to improve such modeling are of critical importance for the advancement of deep learning research in the field. This new architectural approach is easily adaptable to other modeling tasks, forming a valuable foundation for future multimodal modeling research.

## CONFLICT OF INTEREST STATEMENT

The author declares no conflicts of interest.
